# Circulating Diabetic Candidate Neurotrophic Factors, Brain-Derived Neurotrophic Factor and Fibroblast Growth Factor 21, in Sleeve Gastrectomy

**DOI:** 10.1038/s41598-020-62395-z

**Published:** 2020-03-24

**Authors:** Hung-Hsuan Yen, Sung-Tsang Hsieh, Chi-Ling Chen, Wei-Shiung Yang, Po-Chu Lee, Ming-Tsan Lin, Chiung-Nien Chen, Po-Jen Yang

**Affiliations:** 1 Department of Surgery, National Taiwan University Hospital, Taipei, Taiwan; 2 Department of Neurology, National Taiwan University Hospital, Taipei, Taiwan; 3 Department of Anatomy and Cell Biology, College of Medicine, National Taiwan University, Taipei, Taiwan; 4 Graduate Institute of Clinical Medicine, College of Medicine, National Taiwan University, Taipei, Taiwan; 5 Department of Internal Medicine, National Taiwan University Hospital, Taipei, Taiwan; 6 Center for Obesity, Life Style, and Metabolic Surgery, National Taiwan University Hospital, Taipei, Taiwan

**Keywords:** Type 2 diabetes, Obesity

## Abstract

Recent studies show brain-derived neurotrophic factor (BDNF) and fibroblast growth factor 21 (FGF21) are neurotrophic factors associated with obesity and diabetes mellitus (DM). Laparoscopic sleeve gastrectomy (LSG) can significantly reduce weight and improve DM. In this study, we enrolled 78 patients with obesity and evaluated the change of BDNF and FGF21 6 months after LSG. At baseline, the BDNF level was similar between the preoperative DM (n = 30) (17.1 ± 7.7 ng/ml) and non-DM (n = 48) (17.0 ± 6.9 ng/ml) patients with obesity, but FGF21 was significantly higher in the DM patients (201.5 ± 204.3 versus 107.6 ± 63.8 pg/ml). At 6 months after LSG, most of the preoperative DM patients (96.7%) had DM either resolved (66.7%) or improved (30%). BDNF increased and FGF21 decreased significantly regardless of the preoperative DM status, while FGF21 decreased more prominently in the preoperative DM patients (−92.6 ± 179.8 versus −4.6 ± 63.4 pg/ml). After adjusted for age, sex, and preoperative DM status, FGF21 became significantly and positively related to C-peptide (*β* = 18.887), insulin (*β* = 2.399), and homeostasis model assessment of insulin resistance index (*β* = 8.566) after surgery. In conclusion, diabetic patients with obesity had higher FGF21 and similar BDNF levels compared to non-diabetic obese patients. BDNF increased and FGF21 decreased significantly after LSG. FGF21 became positively associated with several insulin-related profiles after surgery.

## Introduction

Laparoscopic sleeve gastrectomy (LSG) has been proven to effectively induce long-term weight loss and alleviate type 2 diabetes mellitus (T2DM) and metabolic diseases in patients with morbid obesity^1^. In addition, several regional and systemic neurologic changes, such as alteration of olfactory and gustatory function^2^, relief of the imbalanced autonomic nervous system^3^, improvement of diabetic neuropathy^4^, and accelerated gastric emptying^5^, have also been observed after LSG.

The mechanisms of metabolic and neurologic changes after bariatric surgery are still under research. Recent proposed hypotheses include the alterations of gut hormones, bile acids, gut microbiota, neural circuits, and neurotrophic factors after bariatric surgery^6–15^. Neurotrophic factors are a family of biomolecules with trophic effects on neurons supporting their growth, survival, and differentiation. Recent studies demonstrated that some neurotrophic factors also act as metabolic regulators. Among all neurotrophic factors, brain-derived neurotrophic factor (BDNF) and fibroblast growth factor 21 (FGF21) are most relevant to obesity, T2DM, and metabolic diseases^7,12,13,16^.

BDNF has been shown to decrease appetite, increase energy expenditure, reduce body weight, and improve hyperglycemic conditions through ameliorating hepatic insulin resistance in animal models^17,18^. BDNF and its receptor have also been found in the central feeding circuits where the injection of BDNF elevated basal metabolic rate, suppressed hyperphagia, and further decreased body weight^19,20^. In human studies, BDNF deficiency or functional impairment contributes to the pathogenesis of obesity, metabolic diseases, insulin resistance, eating disorders, and substance additive disorders^8,21,22^, but the changes in BDNF after weight loss through different weight reduction methods are variable^9–11^.

FGF21, on the other hand, is regarded mostly as a metabolic regulator, but it also exhibits neurotrophic effects^23^. As a metabolic regulator, the secretion of FGF21 is regulated by different dietary composition^24–26^ and serum bile acids level^25,27^, and the increased FGF21 level is addictive to insulin activity to decrease insulin resistance^12,26^. It lowers serum glucose and triglyceride levels via increased glucose transporters GLUT1 expression on adipocytes and reduced glucagon secretion from pancreatic alpha cells^12^. In addition, FGF21 also increases energy expenditure^12,28^, signals to the brain to suppress sugar intake and sweet taste preference^29,30^, and is thus recognized as an atypical neurotrophic factor^23^. A successful reduction in weight and adiposity could be achieved by the injection of FGF21 to the diet-induced obese and *ob/ob* mice; however, the human subject research remains suboptimal^28^. It is known that patients with obesity, T2DM, and metabolic diseases tend to have high FGF21 levels but attenuated FGF21 signaling responses; therefore, previous research has further proposed that obesity, T2DM, and metabolic diseases are in an FGF21-resistant state^13^. Although bariatric surgery can effectively reduce weight and resolve T2DM and metabolic diseases, reports about the changes in FGF21 after bariatric surgery are controversial^14,15,31^.

The existing reports in the literature concerning the relationship between BDNF, FGF21, and bariatric surgery are limited, and the results have been inconclusive^9,11,14,15,31,32^. The current study aimed to investigate the changes in circulating BDNF and FGF21 with weight reduction and the alleviation of metabolic diseases in morbidly obese patients with or without T2DM 6 months after LSG and the associations between these two neurotrophic factors and clinical variables before and after LSG.

## Materials and methods

This is an observational study in a prospectively collected cohort. A total of 78 patients with morbid obesity receiving LSG by a previously described technique^5^ between 2013 and 2017 were included. The diagnosis of T2DM was made based on the criteria provided by American Diabetes Association^33^. Repeated testing was given to the patients with mildly elevated glycosylated hemoglobin (A1C) (6.5–7.0) in the absence of unequivocal hyperglycemia. Patients with previously diagnosed T2DM would keep oral hypoglycemic agents (OHA) or/and insulin injection prescribed by their endocrinologists before surgery. The newly diagnosed T2DM was treated with metformin for at least 1 month prior to the surgery at bariatric surgeon’s clinic. All of the patients gave written informed consent, and the study was approved by the National Taiwan University Hospital Institutional Review Board.

The blood samples were taken before and 6 months after LSG from all of the patients with at least 8-hour fasting. Complete blood cell count, blood chemistry panel, glucose profile, lipid profile, and high-sensitivity C-reactive protein (hsCRP) were all assessed with a standard clinical automatic analyzer. BDNF and total FGF21 were measured using human enzyme-linked immunosorbent assay kits (both R&D Systems, Inc., Minneapolis, MN, USA). Body mass index (BMI), waist circumference (WC), and blood pressure were measured before LSG and at every postoperative outpatient follow-up. The diabetic medications and insulin therapy were tapered off gradually according to the blood glucose levels recorded by the patients at bariatric surgeon’s clinic after LSG.

All of the statistical analyses were performed using R version 3.4.0 (R Foundation for Statistical Computing, Vienna, Austria). The independent and paired two-sample t-tests were used for quantitative variables in two unrelated sample groups and repeated measurements on a single sample group, respectively. The result of quantitative variables was expressed as the mean ± standard deviation. The relationship between BDNF/FGF21 and each individual variable was analyzed by multiple linear regression model adjusted for sex, age, and preoperative DM status and was expressed as regression coefficient. All of the statistical tests were two-sided, and a *P* value <0.05 was considered significant.

### Ethical approval

All procedures performed in studies involving human participants were in accordance with the ethical standards of the institutional and/or national research committee and with the 1964 Helsinki Declaration and its later amendments or comparable ethical standards.

### Informed consent

Informed consent was obtained from all individual participants included in the study.

## Results

The demographic and laboratory data of all of the patients (n = 78) before and 6 months after LSG are shown in Table 1. These patients were further grouped according to the preoperative T2DM status into “preoperative non-DM (n = 48)” and “preoperative DM (n = 30)”, which are also listed in Table 1. The patients in the preoperative DM group were older than those in the non-DM group. The baseline values of blood glucose level, A1C, homeostatic model assessment for insulin resistance (HOMA-IR), alanine aminotransferase (ALT), and triglycerides were all significantly higher in the preoperative DM group. For two neurotrophic factors, BDNF was similar between the two groups while FGF21 was significantly higher in the preoperative DM than in the non-DM group (*P* = 0.020).

**Table 1:** The characteristics of the patients with obesity *before* and *6 months after* laparoscopic sleeve gastrectomy.

	Laparoscopic sleeve gastrectomy (n = 78)					
	All (n = 78)		Preop. Non-DM (n = 48)		Preop. DM (n = 30)	
	Preop.	Postop.	Preop.	Postop.	Preop.	Postop.
Age (year)	37.9 ± 11.1	38.4 ± 11.1	35.9 ± 10.5^*#*^	36.9 ± 10.5	41.0 ± 11.5^*#*^	42.0 ± 11.5
Sex (M/F)	35/43		20/28		15/15	
% EWL		60.3 ± 19.0		61.8 ± 21.1		58.1 ± 15.0
BMI (kg/m^2^)	42.1 ± 6.7	32.0 ± 5.8^*^	42.4 ± 7.6	32.2 ± 6.7^*^	41.6 ± 5.0	31.7 ± 4.0^*^
WC (cm)	121.6 ± 13.6	102.0 ± 14.1^*^	120.6 ± 14.6	101.1 ± 15.7^*^	123.2 ± 12.0	103.5 ± 11.3^*^
SBP (mmHg)	138.9 ± 16.2	125.5 ± 10.9^*^	137.7 ± 17.1	124.3 ± 10.9^*^	140.8 ± 14.7	127.3 ± 10.9^*^
DBP (mmHg)	86.5 ± 12.2	75.5 ± 13.7^*^	86.0 ± 13.2	75.5 ± 10.8^*^	87.4 ± 10.6	75.4 ± 17.6^*^
WBC (k/μL)	8.4 ± 2.2	6.9 ± 1.9^*^	8.3 ± 1.8	6.8 ± 1.9^*^	8.7 ± 2.6	7.2 ± 1.8^*^
Hb (g/dL)	14.7 ± 3.8	13.3 ± 1.7^*^	14.4 ± 1.4	13.6 ± 1.3^*^	14.2 ± 1.7	12.9 ± 2.2^*^
C-peptide (ng/mL)	4.3 ± 1.8	2.2 ± 0.7^*^	4.2 ± 1.7	2.2 ± 0.7^*^	4.5 ± 2.2	2.2 ± 0.8^*^
Insulin (μIU/mL)	29.2 ± 18.3	9.8 ± 5.2^*^	26.4 ± 15.4	9.5 ± 5.3^*^	33.9 ± 21.7	10.3 ± 5.2^*^
Blood glucose (mg/dL)	113.9 ± 44.6	92.3 ± 21.0^*^	92.7 ± 7.8^*#*^	85.2 ± 6.0^*,*#*^	149.0 ± 57.0^*#*^	103.5 ± 30.0^*,*#*^
A1C (%)	6.6 ± 1.4	5.5 ± 0.7^*^	5.8 ± 0.4^*#*^	5.3 ± 0.3^*,#^	8.0 ± 1.3^#^	5.9 ± 0.9^*,*#*^
HOMA-IR	8.2 ± 6.3	2.3 ± 1.4^*^	6.1 ± 3.8^*#*^	2.0 ± 1.2^*^	11.8 ± 8.0^#^	2.7 ± 1.6^*^
ALT (U/L)	44.8 ± 29.3	17.9 ± 14.9^*^	38.7 ± 21.5^*#*^	17.0 ± 11.5^*^	54.4 ± 37.1^*#*^	19.3 ± 19.3^*^
Triglyceride (mg/dL)	187.4 ± 111.0	102.1 ± 38.4^*^	155.1 ± 75.3^*#*^	91.7 ± 28.2^*,#^	239.1 ± 137.9^*#*^	118.6 ± 46.5^*,*#*^
Total cholesterol (mg/dL)	188.9 ± 34.5	185.3 ± 32.6	193.1 ± 34.9	189.8 ± 33.5	182.2 ± 33.4	178.0 ± 30.1
HDL cholesterol (mg/dL)	42.3 ± 8.9	47.0 ± 11.5^*^	42.9 ± 8.7	48.7 ± 12.6^*^	41.4 ± 9.4	44.2 ± 9.0^*^
hsCRP (mg/dL)	0.7 ± 0.6	0.4 ± 0.5^*^	0.7 ± 0.6	0.4 ± 0.6^*^	0.7 ± 0.5	0.4 ± 0.3^*^
BDNF (ng/ml)	17.0 ± 7.4	20.6 ± 10.0^*^	17.1 ± 7.7	22.8 ± 11.0^*^	17.0 ± 6.9	22.3 ± 8.2^*^
FGF21 (pg/ml)	143.7 ± 142.6	105.8 ± 52.6^*^	107.6 ± 63.8^*#*^	103.8 ± 50.4^*^	201.5 ± 204.3^*#*^	109.0 ± 56.7^*^

The data are mean ± standard deviation.

DM = diabetes mellitus; Preop. = preoperative; Postop. = postoperative (6 months); % EWL = percentage of excess weight loss; BMI = body mass index; WC = waist circumference; SBP = systolic blood pressure; DBP = diastolic blood pressure; WBC = white blood cell; Hb = hemoglobin; A1C = glycosylated hemoglobin; HOMA-IR = homeostatic model assessment for insulin resistance; ALT = alanine transaminase; HDL = high-density lipoprotein; hsCRP = high-sensitivity C-reactive protein; BDNF = brain-derived neurotrophic factor; FGF21 = fibroblast growth factor 21.

^*^P value < 0.05 after comparing between preoperative and postoperative variables.

^*#*^*P* value < 0.05 after comparing preoperative or postoperative variables between preoperative DM and preoperative non-DM.

Six months after LSG, 20 of 30 (66.7%) preoperative DM patients had normal glycemic profiles without any antidiabetic medication, and 9 of 30 (30%) had improvements in T2DM. Only 1 of 30 (3.3%) had persistent T2DM. BMI, WC, systolic blood pressure (SBP), diastolic blood pressure (DBP), white blood cell (WBC), hemoglobin (Hb), C-peptide, insulin, blood glucose, A1C, HOMA-IR, ALT, triglyceride, and hsCRP decreased significantly in all of the patients irrespective of their preoperative DM status. The high-density lipoprotein cholesterol concentration, on the other hand, significantly increased 6 months after LSG (Table 1). The percentage of excess weight loss was similar between the preoperative non-DM and DM patients (*P* = 0.370). The increases in the BDNF level were also similar between the preoperative non-DM and DM patients (5.9 ± 12.3 versus 5.4 ± 8.9 ng/ml; *P* = 0.842). On the contrary, FGF21 decreased more significantly in the preoperative DM (−92.6 ± 179.8 pg/ml) than the non-DM (−4.6 ± 63.4 pg/ml) patients (*P* = 0.015).

Table 2 summarizes the multiple linear regression model of BDNF/FGF21 and each individual variable at baseline and 6 months after LSG, after sex, age, and preoperative DM status were adjusted. BDNF was positively related to WBC either at baseline (*P* = 0.028) or 6 months after LSG (*P* = 0.024) in all of the patients. On the other hand, BDNF was positively related to total cholesterol (*P* = 0.033) but negatively associated with ALT (*P* = 0.012) only at 6 months after LSG. There was no significant relationship between FGF21 and each other individual variable at baseline, while 6 months after LSG, FGF21 was positively related to C-peptide (*P* = 0.005), insulin (*P* = 0.029), HOMA-IR (*P* = 0.044), and hsCRP (*P* < 0.001) and negatively related to total cholesterol (*P* = 0.025).

**Table 2:** Multiple linear regression analyses (adjusted for sex, age, and preoperative type 2 diabetes mellitus status) between the circulating levels of BDNF/ FGF21 and each indicated variable.

*β*	BDNF		FGF21	
	Preop. (n = 78)	Postop. (n = 78)	Preop. (n = 78)	Postop. (n = 78)
BMI	−0.046	−0.236	0.306	1.019
WBC	0.968^*^	1.479^*^	6.039	−0.617
Blood glucose	−0.013	0.032	−0.227	0.171
C-peptide	0.047	1.100	10.929	18.887^*^
Insulin	−0.021	0.029	0.787	2.399^*^
A1C	−0.415	0.876	−18.367	5.229
HOMA-IR	−0.026	0.282	1.849	8.566^*^
ALT	−0.007	−0.198^*^	−0.022	−0.550
Triglyceride	−0.014	0.011	−0.002	0.219
Total cholesterol	−0.015	0.077^*^	−0.076	−0.392^*^
HDL cholesterol	0.002	−0.091	0.188	−1.027
hsCRP	0.194	0.287	19.450	42.642^*^

*β* = The regression coefficients for each indicated variable adjusted for sex, age, and preoperative type 2 diabetes mellitus status.

FGF21 = fibroblast growth factor 21; BDNF = brain-derived neurotrophic factor; Preop. = preoperative; Postop. = postoperative (6 months); BMI = body mass index; WBC = white blood cell; A1C = glycosylated hemoglobin; HOMA-IR = homeostatic model assessment for insulin resistance; ALT = alanine transaminase; HDL = high-density lipoprotein; hsCRP = high-sensitivity C-reactive protein.

^*^P value < 0.05.

## Discussion

BDNF and FGF21 have been implicated in the pathogenesis of obesity, T2DM, and metabolic diseases. Many studies have attempted to document the mechanism and develop a new treatment^7,13,16,17,28,29,34^. Previous studies focused on the changes in BDNF and FGF21 in patients with obesity treated with a hypocaloric diet^8,13,15^, exercise^10^, or different types of bariatric surgery^9,11,14,15,31,32^; nevertheless, LSG has been less frequently studied. The current report found that BDNF increased significantly while FGF21 decreased significantly 6 months after LSG, regardless of the preoperative DM status. The baseline BDNF levels were similar between the preoperative non-DM and DM patients; however, the baseline FGF21 level was significantly higher in the preoperative DM patients. More decrease in the circulating levels of FGF21 was observed in the preoperative DM patients than in the preoperative non-DM patients 6 months after LSG. No significant relationships related to FGF21 were found at baseline; nevertheless, FGF21 was positively related to C-peptide, insulin, HOMA-IR, and hsCRP and negatively related to total cholesterol 6 months after LSG.

The physiological role of BDNF includes appetite control, energy expenditure, and glucose homeostasis^17,18^. Kernie *et al*.^17^ found that BDNF mutant heterozygous mice were hyperphagic, obese, and prone to developing insulin resistance, dyslipidemia, and hyperglycemia. Furthermore, infusing these mice with BDNF could transiently reverse the eating behavior and obesity^17^. In a human study, de Luis *et al*.^8^ revealed less decrease in BMI, WC, triglyceride, and HOMA-IR in patients with the T allele of rs10767664, a single-nucleotide polymorphism of the BDNF gene, after weight reduction via a hypocaloric diet. These studies^8,17^ imply that either BDNF deficiency or functional impairment could contribute to the pathogenesis of obesity, insulin resistance, and metabolic diseases.

In line with our findings, Roh *et al*.^10^ found that the circulating BDNF level increased after 8 weeks of aerobic exercise-induced weight loss in men with obesity. On the contrary, Merhi *et al*.^9^ found significantly decreased BDNF levels after Roux-en-Y gastric bypass (RYGB) and gastric banding in a small patient group inclusive of either pre- (n = 12) or postmenopausal women (n = 6). Muñoz-Rodríguez *et al*.^11^ also found significantly decreased BDNF one year after RYGB in 27 patients with morbid obesity. In fact, the level of BDNF is affected by fasting condition upon blood draw, different phases of the menstrual cycle^9^, several diseases, and medications. Despite different measurement methods (8-hour fasting, 6 months after LSG), ethnic group (Asian), and more male patients included in our study, the most significant difference between the current study and those by Merhi *et al*.^9^ and Muñoz-Rodríguez *et al*.^11^ was that a single bariatric surgery type, LSG, was used in all of our patients. Unlike LSG, RYGB modifies the route of food transition and increases the serum bile acids levels in enterohepatic circulation^35^, which, mediate the metabolic change, and possibly, contribute to the decreased BDNF levels postoperatively.

Obesity has been described as an FGF21-resistant state because elevated circulating levels of FGF21 and attenuating FGF21 signaling responses with an impaired reduction in circulating glucose and lipid were observed in diet-induced obese mice^13^. Tanajak *et al*. further revealed the elevation of FGF21 appeared prior to the development of insulin resistance and dyslipidemia in high-fat diet-induced obese rats^34^. In our study, the FGF21 level was significantly higher in the preoperative DM patients at baseline and decreased a greater amount 6 months after surgery compared to the preoperative non-DM patients. Furthermore, FGF21 was not related to any metabolic parameters, regardless of sex, age, or preoperative DM status at baseline; however, FGF21 was positively related to C-peptide, insulin, HOMA-IR, and hsCRP and negatively related to total cholesterol 6 months after LSG. The metabolic effect of FGF21 might be attenuated by morbid obesity at baseline; nevertheless, after the excess weight was reduced, the association between FGF21 and insulin secretion was restored. This finding implies that the patients with morbid obesity had reversed from an FGF21-resistant state 6 months after LSG, and potentially, the FGF21 level might serve as an early predictor for recovery from morbid obesity, T2DM, and metabolic diseases. On the other hand, the therapeutic role of FGF21 for obesity was evaluated in an animal study. Coskun *et al*. reported a reduction in weight and adiposity in diet-induced obese and *ob/ob* mice after a 2-week systemic injection of FGF21. No decrease in total caloric intake or effect on physical activity was observed, but the FGF21-treated mice exhibited increased energy expenditure, fat utilization, and lipid excretion, reduced hepatosteatosis, and ameliorated glycemia^28^. Nevertheless, whether this therapeutic approach is also effective in human subjects and the selection criteria for whom would benefit from the FGF21 treatment require further investigations.

RYGB and LSG have different impacts on the circulating level of FGF21. Lips *et al*. found the total FGF21 and serum bile acids levels decreased at 3 weeks and 3 months after long-term calorie restriction by either gastric banding or a very-low-calorie diet but increased after RYGB in females with morbid obesity^15^. Crujeiras *et al*. found increased total FGF21 levels at 6 or 12 months after bariatric surgery with different types of bariatric surgery included in their study and RYGB accounting for the most (LSG 8.3%, RYGB 54.2%, and biliopancreatic diversion 37.5%)^14^. Woelnerhanssen *et al*. revealed no changes in intact FGF21 levels at 12 months after RYGB or LSG^31^. In our study, we collected a relatively larger number of patients and demonstrated a significantly lower total FGF21 level at 6 months after LSG. These discrepancies in the literatures resulted from the small number of study patients, the timing (durations after surgery) and method (total or intact form of FGF21) for FGF21 measurement, and various types (RYGB or LSG) of bariatric surgery.

The FGF21 secretion is regulated by dietary composition and serum bile acids level^24–27^. A low protein, high carbohydrate diet elevates the FGF21 level via carbohydrate-response element-binding protein^26^, and a high fat, low carbohydrate ketogenic diet activates peroxisome proliferator-activated receptor-alpha (PPAR-α) and then increases FGF21 gene expression^24,25^. On the other hand, serum bile acids induce FGF21 gene expression through the activation of Farnesoid X receptor pathway^25,27^. All of our patients were educated on increasing dietary protein intake and decreasing high-carbohydrate beverages after surgery, and most of them had been strictly followed in a dietitian’s clinic. Not only the gastric volume but also the dietary composition was changed in our patients after LSG. Moreover, different bariatric surgery procedures lead to the different changes in serum bile acids level after surgery^35–37^. The serum bile acids level was significantly decreased 12 months after LSG according to De Vuono *et al*.^37^ but was significantly increased after RYGB^35,36^. Besides, RYGB rearranges the gastrointestinal tract, increases gastric emptying, decreases nutrient absorption, and causes rapid elevation of blood glucose and free fatty acid levels simultaneously. These differences might explain the opposite changes of FGF21 levels after surgery between LSG and RYGB^14,15,32^.

The strength of our study is that we compared the circulating BDNF/FGF21, clinical characteristics, and biochemical parameters in patients with morbid obesity before and 6 months after LSG. To the best of our knowledge, this is the first study to demonstrate the relationship between circulating BDNF/FGF21 and LSG. We also evaluated the differences based on the preoperative DM status. However, this study had several limitations. The number of patients included was relatively small, and the measurements of circulating BDNF and FGF21 were taken only twice (before and 6 months after LSG) in a fasting condition. Moreover, the total FGF21 measured in our study may not represent the metabolic potential of the FGF21 because only the intact form of the FGF21 is functional^38^. In addition, the lack of adequate control groups, including healthy (non-obese), non-surgical weight loss (hypocaloric diet or exercise), and different surgical weight loss (gastric bypass) control groups, limited our comparison and implication. The standard detection method and reference range for BDNF and FGF21 have not been well-stablished, so further *in vitro* and *in vivo* studies will be needed to clarify the exact roles of BDNF and FGF21 in metabolism and their possible application in clinical practice.

## Conclusions

Different neurotrophic factors have varying diabetic and metabolic effects. The circulating level of FGF21 was higher in the diabetic patients with morbid obesity than the non-diabetic subjects, while the level of BDNF was similar between the two groups. BDNF increased and FGF21 decreased significantly 6 months after LSG. In addition, the associations between FGF21 and several insulin-related profiles, including C-peptide, insulin, and HOMA-IR, after LSG were not noted in the patients with morbid obesity before surgery.
